# Cryptolepine-Induced Cell Death of *Leishmania donovani* Promastigotes Is Augmented by Inhibition of Autophagy

**DOI:** 10.4061/2011/187850

**Published:** 2011-03-03

**Authors:** Souvik Sengupta, Sayan Chowdhury, Somdeb BoseDasgupta, Colin W. Wright, Hemanta K. Majumder

**Affiliations:** ^1^Molecular Parasitology Laboratory, Indian Institute of Chemical Biology, 4, Raja S. C. Mullick Road, Jadavpur, Kolkata 700032, India; ^2^Infection Biology, Biozentrum, University of Basel, Klingelbergstrasse 50/70, 4056 Basel, Switzerland; ^3^Bradford School of Pharmacy, University of Bradford, West Yorkshire BD7 1DP, UK

## Abstract

*Leishmania donovani* are the causative agents of visceral leishmaniasis worldwide. Lack of vaccines and emergence of drug resistance warrants the need for improved drug therapy and newer therapeutic intervention strategies against leishmaniasis. In the present study, we have investigated the effect of the natural indoloquinoline alkaloid cryptolepine on *L. donovani* AG83 promastigotes. Our results show that cryptolepine induces cellular dysfunction in *L. donovani* promastigotes, which leads to the death of this unicellular parasite. Interestingly, our study suggest that cryptolepine-induced cell death of *L. donovani* is counteracted by initial autophagic features elicited by the cells. For the first time, we show that autophagy serves as a survival mechanism in response to cryptolepine treatment in *L. donovani* promastigotes and inhibition of autophagy causes an early increase in the amount of cell death. This study can be exploited for designing better drugs and better therapeutic strategies against leishmaniasis in future.

## 1. Introduction

Species of the genus *Leishmania *are the causative agents of various parasitic infections which manifest itself in a variety of clinical forms depending upon the species of *Leishmania* and the immunological status of the host. *Leishmania donovani* is the causative agent of visceral leishmaniasis (VL) or “Kala-azar”, which is fatal if patients are left untreated and is more common in less developed countries [1]. The organism has a digenic life cycle residing as flagellated extracellular promastigotes in the gut of insect vector and as nonflagellated amastigotes in mammalian host macrophages [2]. The drugs recommended for treatment of VL, namely, pentavalent antimonials, amphotericin B, and lipid formulations of amphotericin B, have many limitations like long course of treatment, toxic side effects and high costs [1]. Moreover, the occurrence of chemoresistance against classical drugs has worsened the situation further [3, 4]. Thus, search for new drugs, new molecular targets, and novel therapeutic strategies are justified.

In search of better leishmanicidal compounds, plant derived compounds have long been evaluated [5]. There has been considerable public and scientific interest in the use of plant derived compounds to combat human diseases. Cryptolepine is an indoloquinoline alkaloid which was first isolated from the roots of *Cryptolepis triangularis *collected in Belgian Congo and afterward from the roots of *Cryptolepis sanguinolenta *from Nigeria [6]. This species has been used traditionally to treat malaria, hypertension, hyperglycemia, inflammation and cancer [7, 8]. Although there are reports of antiparasitic activity of CLP [9], its effect on *Leishmania donovani* is yet to be evaluated.

Programmed cell death (PCD) appears to be the most preferred mechanism for mediating parasiticidal activity, as has been observed in kinetoplastids in response to diverse stimuli, for example, heat shock, chemotherapeutic agents such as pentostam, amphotericin B [10], camptothecin [4], oxidants such as H_2_O_2_ [11] or even serum deprivation [12]. Apoptosis involves a series of morphological and biological changes including ROS production, decrease in cellular GSH levels, and so forth, which ultimately results in DNA fragmentation [3, 4, 13]. This is considered as the hallmark of apoptosis. However, it has also been suggested that autophagy provides the front line of defense against oxidative stress [14] and can actually protect cells by preventing them from undergoing apoptosis [15]. Autophagy is an evolutionarily conserved mechanism for the degradation of cellular components in the cytoplasm [16] resulting in eventual breakdown and recycling of macromolecules [17]. Though autophagic cell death has been suggested to be involved in various systems [18], the precise role of this catabolic process in dying cells is not clear [16]. In fact, autophagy may have originally arisen as a mechanism to protect unicellular organisms against any form of environmental stress [19]. Autophagy plays a role in lifespan extension and Sir2 has been suggested to be involved in the process [20]. However, role of autophagy as a survival mechanism in response to drug in *Leishmania* remains to be elucidated.

In the present study, we have evaluated the effect of naturally occurring indoloquinoline alkaloid cryptolepine (CLP) on *L. donovani* AG83 promastigotes. We show that CLP induces ROS in the cells, ultimately resulting in DNA fragmentation which is a hallmark of apoptosis. For the first time, we identified that the parasites try to combat against initial CLP-induced stress response by initiating an autophagic response as a survival mechanism and activation of silent information regulator protein Sir2 plays a role in the process. This study has a great potential in understanding the role of autophagy in the cell death mechanism of *Leishmania* and will be helpful in identifying new drugs and newer therapeutic strategies to combat leishmaniasis in future.

## 2. Materials and Methods

### 2.1. Chemicals

**Figure 1:**
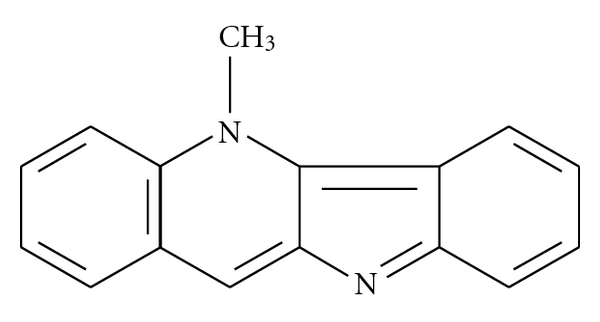
Structure of Cryptolepine.

Cryptolepine (Figure 1) hydrochloride was synthesized using isatin and O, N-acetylindoxyl as described previously [21] and was dissolved at 20 mM concentration in 100% DMSO and stored at −20°C. *N*-Acetyl-L-cysteine (NAC) was purchased from Sigma-Aldrich, was dissolved in 100% DMSO at 50 mM and stored at −20°C. FM4-64 and monodansylcadaverine (MDC) and monochlorobimane were purchased from Molecular Probes and stored at −20°C and room temperature, respectively.

### 2.2. Parasite Culture and Maintenance

The *L*. *donovani *strain AG83 promastigotes were grown at 22°C in Ray's modified media [22] and in M199 liquid media supplemented with 10% fetal calf serum as described previously [3].

### 2.3. Cell Viability Test by MTT Assay

The effect of drug on the viability of *L. donovani *AG83 promastigote cells was determined by 3-(4,5-dimethylthiazol-2-yl)-2,5-diphenylterazolium bromide (MTT) assay [2, 23]. The cells at the exponential phase were collected and transferred into 24-well plate (approximately 4 × 10^6^ cells/well). The cells were then incubated for various time periods in the presence of different concentrations of cryptolepine hydrochloride (CLP). After incubation, the cells were centrifuged and the supernatant was aspirated. The cell pellet was washed with PBS (1X) twice and then finally suspended in 100 *μ*L of PBS (1X) in 96-well plates. Ten microliters of MTT solution (10 *μ*g/mL) were added in each sample of 96-well plates and samples were incubated for 4 h. After incubation, 100 *μ*L of stop solution (stock: 4963 *μ*L of isopropanol and 17 *μ*L of concentrated HCl) was added and kept for 20 min at room temperature. The optical density was taken at A_570_ on an ELISA reader (Multiskan EX; Thermo Fisher Scientific, Waltham, MA). 

### 2.4. Study of Parasite Ultrastructure by Transmission Electron Microscopy

Transmission electron microscopy (TEM) was carried out with both CLP treated and untreated cells as described previously [4, 13]. Sections were cut with a Du-point diamond knife in an LKB Ultramicrotome, stained on copper grids with uranyl acetate and lead acetate for 10–15 min, respectively, and examined under JEOL 100CX TEM.

### 2.5. Double Staining and Confocal Microscopy

*L. donovani* AG83 promastigotes (approximately 10^6^ cells/mL) were cultured in 24-well plates with different treatments. FM4-64 (40 *μ*M) (Excitation wavelength = 505 nm, emission wavelength = 725 nm) was added directly in the culture medium and kept for 90 min at room temperature. The cells were then washed twice with 1X PBS and stained with 50 *μ*M MDC (Excitation wavelength = 335 nm, emission wavelength = 518 nm) for 10 min at room temperature. The cells were further washed twice with 1X PBS and live promastigotes were immobilized by mounting under poly-L-lysine coated coverslips as described previously [24]. Samples were viewed with a Nikon A1 R laser confocal microscope.

### 2.6. Measurement of Intracellular ROS Levels

Intracellular ROS level was measured in CLP-treated and untreated leishmanial cells as described previously [3]. In brief, after treatment with CLP and NAC for different time periods, cells (approximately 10^6^) were washed and resuspended in 500 *μ*L of medium 199 and were then loaded with a cell-permeate probe CM-H_2_DCFDA for 1 h. This is a nonpolar compound that is hydrolyzed within the cell to form a nonfluorescent derivative, which in presence of a proper oxidant converted to a fluorescent product. Fluorescence was measured through spectrofluorometer using 507 nm as excitation and 530 nm as emission wavelengths.

### 2.7. Measurement of GSH Level

GSH level was measured by monochlorobimane dye that gives a blue fluorescence when bound to glutathione [2, 3]. *L. donovani *promastigotes (approximately 10^6^ cells) were treated with or without CLP at different times. The cells were then pelleted down and lysed by cell lysis buffer according to the manufacturer's protocol (ApoAlert glutathione assay kit; Clontech, Mountain View, CA). Cell lysates were incubated with monochlorobimane (2 mM) for 3 h at 37°C. The decrease in glutathione levels in the extracts of nonapoptotic and apoptotic cells were detected by spectrofluorometer with 395-nm excitation and 480-nm emission wavelengths.

### 2.8. Measurement of Total Fluorescent Lipid Peroxidation Product

CLP-treated and -untreated *L. donovani *cells were pelleted down and washed twice with 1X PBS. The pellet was dissolved in 2 mL of 15% SDS-PBS solution. The fluorescence intensities of the total fluorescent lipid peroxidation products were measured with excitation at 360 nm and emission at 430 nm as described previously [3, 4].

### 2.9. Flow Cytometric Analysis

The *L. donovani *promastigotes were treated with CLP at 20 *μ*M and 3-methyladenine (3-MA) at 10 mM for different times and washed twice with PBS. The cells were then resuspended in 100 *μ*L of binding buffer provided with the FLUOS-annexinV staining kit (Roche Diagnostics). The cells were stained with annexin V-FITC and PI as per instructions given by the manufacturer, and then they were scanned for fluorescence intensity of cell population in different quadrants. The fraction of cell population in different quadrants was analyzed using quadrant statistics [3, 13]. Cells treated with 3-MA alone served as the control for the experiment.

### 2.10. Real-Time PCR Analysis

Total RNA was prepared from *L. donovani* AG83 promastigotes after different treatments for different times using the Total RNA isolation kit (Roche Biochemicals). cDNA was synthesized from 60 ng of total RNA using Superscript II RNaseH^−^ Reverse Transcriptase (Invitrogen) and oligo (dT)_12−18_ primers (Invitrogen) following manufacturers instructions. For amplification of the desired cDNA, gene-specific primers were designed from sequencing data bank website (Table 1). Real-Time PCR was performed for ATG 8, Sir2, and GAPDH genes. Three separate reactions were carried out using three different RNA preparations in 25 *μ*L volume using SYBR-Green Super mix (Applied Biosystem) and same primer sets in a 7300 Real-Time PCR system (Applied Biosystem). Reactions were carried out using the following profile: initial denaturation at 95°C for 5 min followed by 35 cycles with denaturation at 95°C for 45 s, annealing at 52°C for 45 s and extension at 68°C for 45 s. The PCR was followed by a melt curve analysis to ascertain that the expected products were amplified. Values for each gene were normalized to expression levels of GAPDH using the 2^−∆∆*Ct *^ method [25, 26]. The fold expression was calculated as described previously [25, 26] using the following equation:

(1) $$\text{Fold}\;\text{expression}=2^{-∆∆Ct}.$$

### 2.11. DNA Fragmentation Assay

The assay was performed as described previously [4, 13]. Briefly, genomic DNA was isolated from the parasites (approximately 10^6^ cells/mL) after different treatments using an apoptotic DNA ladder kit (Roche Diagnostics). The DNA was quantified and equivalent amount of DNA was electrophoresed in a 1.5% agarose gel at 75 V for 2 h and thereafter stained with EtBr and photographed under UV illumination. 

## 3. Results

### 3.1. Cryptolepine (CLP) Causes Loss of Cell Viability of *L. donovani* Promastigotes

**Figure 2:**
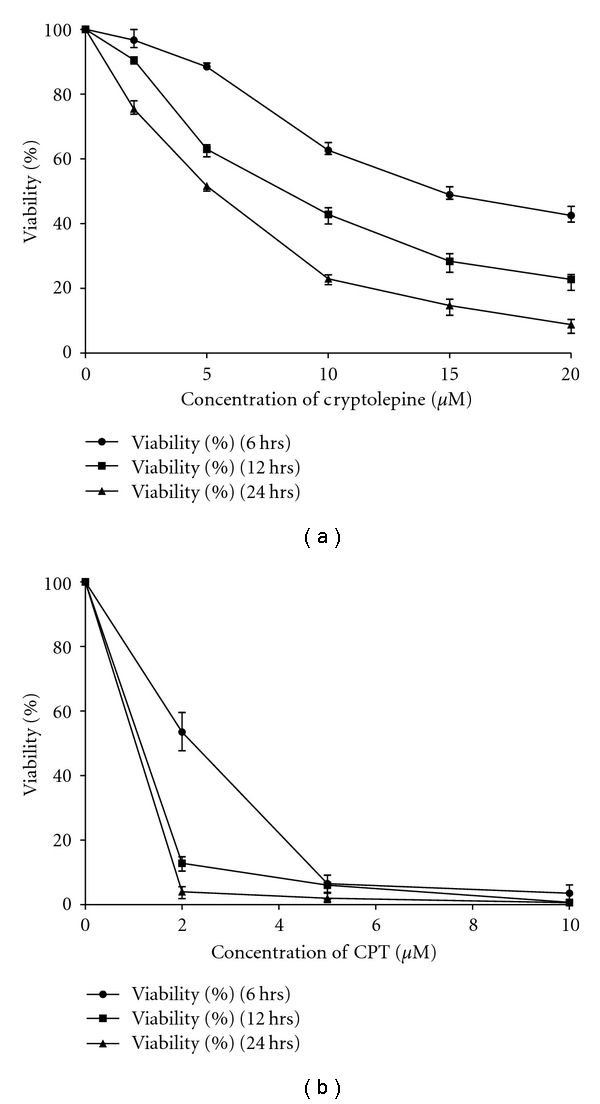
Measurement of cell viability by MTT assay. Log phase *L. donovani* AG83 promastigote cells (4 × 10^6^ cells/mL) were treated with different concentrations of CLP (2, 5, 10, 15, and 20 *μ*M) for different time periods (6, 12, and 24 h) (a) and CPT (2, 5, and 10 *μ*M) for different time periods (6, 12, and 24 h) (b) and percentage of cell viability was measured by MTT assay. Data are represented as Mean ± SEM (*n* = 3).

*L. donovani* AG83 promastigotes (4 × 10^6^ cells/mL) were incubated with five different concentrations of CLP (2, 5, 10, 15, and 20 *μ*M) for 6, 12, and 24 h after which the cell viability was determined by MTT assay (Figure 2(a)). At 12 h, 80% growth was inhibited by 20 *μ*M CLP which was comparable with the inhibition achieved by 10 *μ*M CLP at 24 h and 92% growth was inhibited by 20 *μ*M CLP at 24 h. The effect of CLP was to cause both time- and concentration-dependent decrease in cell viability of *L. donovani* promastigotes. The IC_50_ value of CLP was calculated to be 8.2 *μ*M at 12 h in *L. donovani* AG83 promastigotes. As a positive control, cells were treated with different concentrations of camptothecin (CPT) (2, 5, and 10 *μ*M) for 6, 12, and 24 h and cell viability was determined by MTT assay (Figure 2(b)).

### 3.2. Parasite Ultrastructural Studies Using Transmission Electron Microscopy

**Figure 3:**
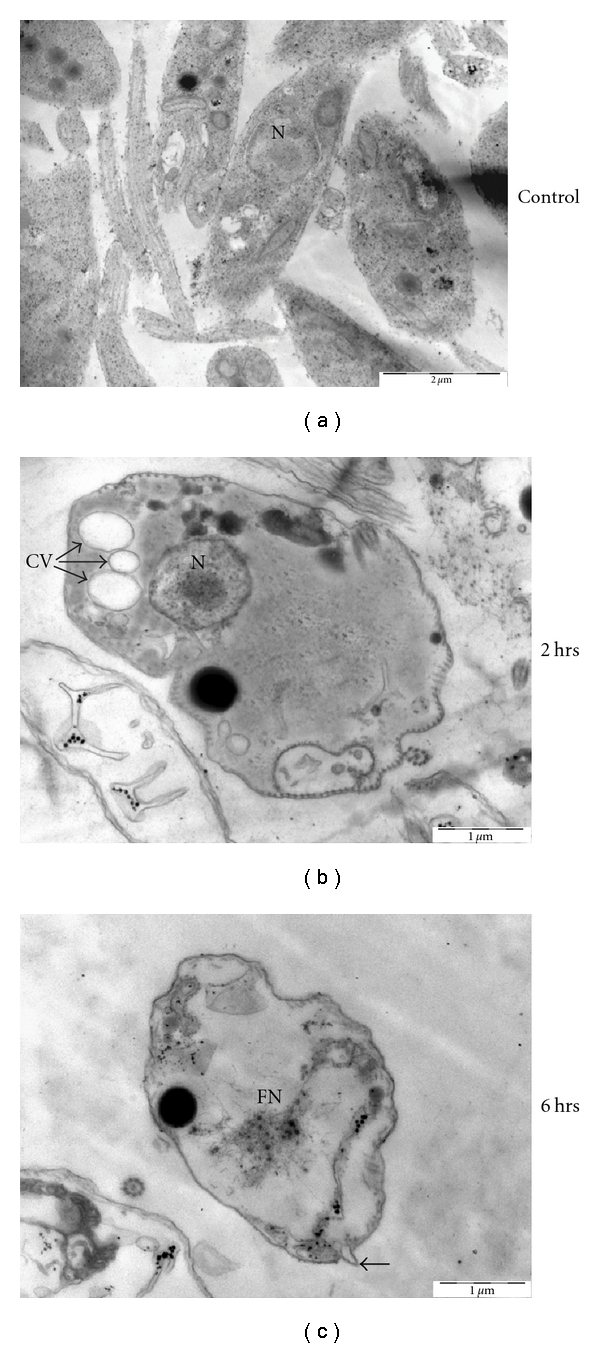
Electron microscopic analysis of *L. donovani* AG83 cells treated with 0.2% DMSO alone and 20 *μ*M CLP for different time periods. Spur blocks were prepared as described in Section 2. (a) Control cells treated with 0.2% DMSO alone, (b) cells treated with 20 *μ*M CLP for 2 h, and (c) cells treated with 20 *μ*M CLP for 6 h. Scale bars are indicated in the figure. N: nucleus, CV: cytoplasmic vacuoles, FN: fragmented nucleus, and closed arrow represents membrane blebbing.

To understand the effect of CLP on *L. donovani* promastigotes in detail, we carried out transmission electron microscopy (TEM) with CLP-treated and -untreated cells for different time points. DMSO treated parasites (control cells) retain the normal nuclear architecture with a prominent central or slightly eccentrically localized nucleolus, while chromatin was usually distributed peripherally beneath the nuclear membrane (Figure 3(a)). Treatment with CLP for 2 h revealed the appearance of multiple cytoplasmic vacuoles, but the nucleus appeared normal with minimum evidence of chromatin condensation. There is also one mitochondrion profile which is swollen, and the matrix appears to be lost (Figure 3(b)). However, treatment with CLP for 6 h causes extensive damage to the cells. The cells exhibited condensed and marginated chromatin and fragmented nucleus. The integrity of the plasma membrane was apparently maintained and membrane blebbing was also observed (Figure 3(c)). Taken together, these results suggest the involvement of initial autophagic response on treatment of *L. donovani *promastigotes with CLP. However, at a later time period, cells exhibit features of apoptotic like cell death.

### 3.3. Double Staining with MDC and FM4-64

**Figure 4:**
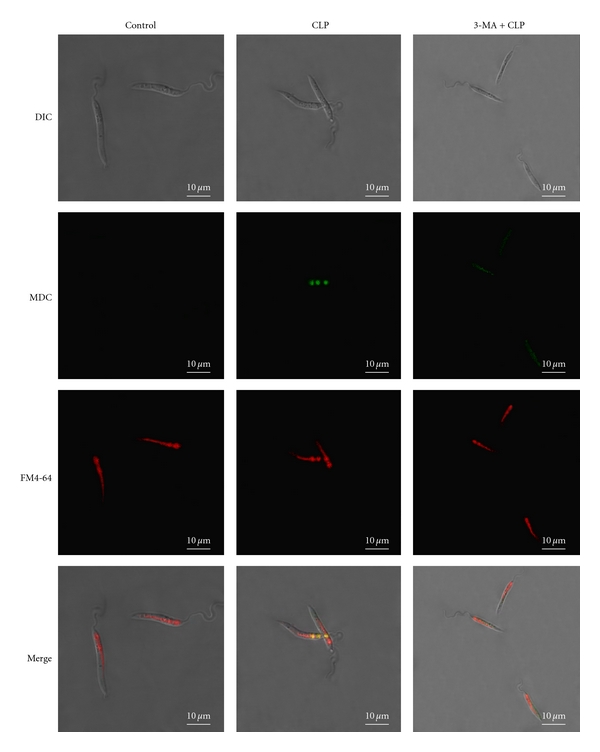
Double staining with FM4-64 and MDC. Slides were prepared as described in Section 2. Confocal microscopic photographs (100X) of control cells, cells treated with CLP (20 *μ*M) for 2 h and cells pretreated with 3-MA (10 mM) and then treated with CLP (20 *μ*M) are shown. DIC denotes differential interference contrast image. FM4-64 signal is shown in red and MDC signal is shown in green. Colocalization of these two markers is shown in yellow. Scale bar is as indicated in the figure.

To confirm the formation of autophagic vacuoles, we next carried out staining with monodansylcadaverine (MDC). MDC is an autofluorescent, autophagolysosome marker that specifically labels autophagic vacuoles *in vivo* and *in vitro* conditions [27–30]. The autophagic machinery involves the fusion of the autophagic vacuoles with the lysosomal compartment for degradation [31]. The lipophilic dye FM4-64 is a fluorescent endocytic marker which has been used in *Leishmania* as a marker for the MVT-lysosome [24, 32]. FM4-64 was found to localize in a tubular compartment in control cells and no fluorescence of MDC was observed under these conditions. However, upon treatment with 20 *μ*M CLP for 2 h, MDC labeled vesicles were observed which colocalized with FM4-64 labelled compartment (Figure 4). Moreover, pretreatment of cells with 3-methyladenine (3-MA), a specific inhibitor of autophagy [33, 34], caused disappearance of MDC labelled vesicles with no change in FM4-64 labelling pattern. Altogether, these observations suggest the involvement of autophagy in response to CLP treatment.

### 3.4. CLP Induces the Formation of ROS inside the Cells Resulting in Cellular Oxidative Stress

**Figure 5:**
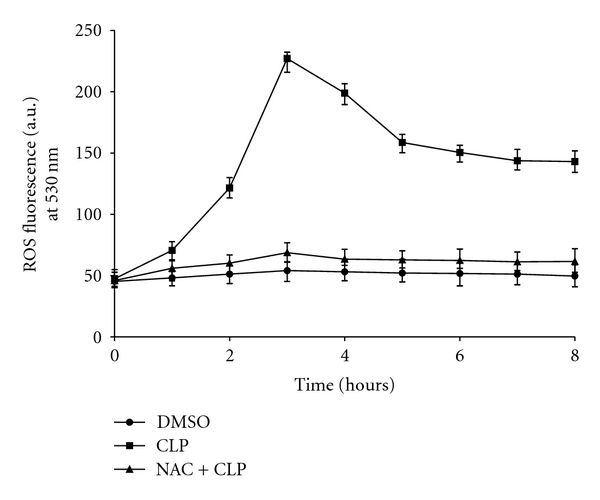
Measurement of CLP-induced generation of ROS. Cells were treated with 20 *μ*M of CLP for different time periods as described in Section 2. Generation of ROS inside the cells was measured after treatment with 0.2% DMSO alone (closed circles), CLP (closed squares) and with NAC prior to treatment with CLP (closed triangles). Data are represented as Mean ± SEM (*n* = 3).

The results of the EM study suggested that apoptotic like cell death might be occurring in CLP treated parasites at a later time point. A key regulator for induction of apoptosis is intracellular ROS [3, 4]. So, next we wanted to see if CLP causes generation of ROS inside the cells. To measure the status of ROS inside cells, we used a spectrofluorometric assay using CM-H_2_DCFDA as described in Section 2. DMSO treated cells (control cells) contained a basal level of ROS whereas treatment with 20 *μ*M CLP caused a 4-fold increase in the ROS levels in parasites at 3 h time period (Figure 5). When cells were pretreated with NAC (20 mM), the level of ROS generation decreased and was nearly same as that of control cells. Thus, it is conceivable from the above result that CLP causes oxidative stress in *Leishmania* parasites.

### 3.5. CLP-Induced Oxidative Stress Causes Depletion of Cellular GSH Level and Increases the Level of Lipid Peroxidation

**Figure 6:**
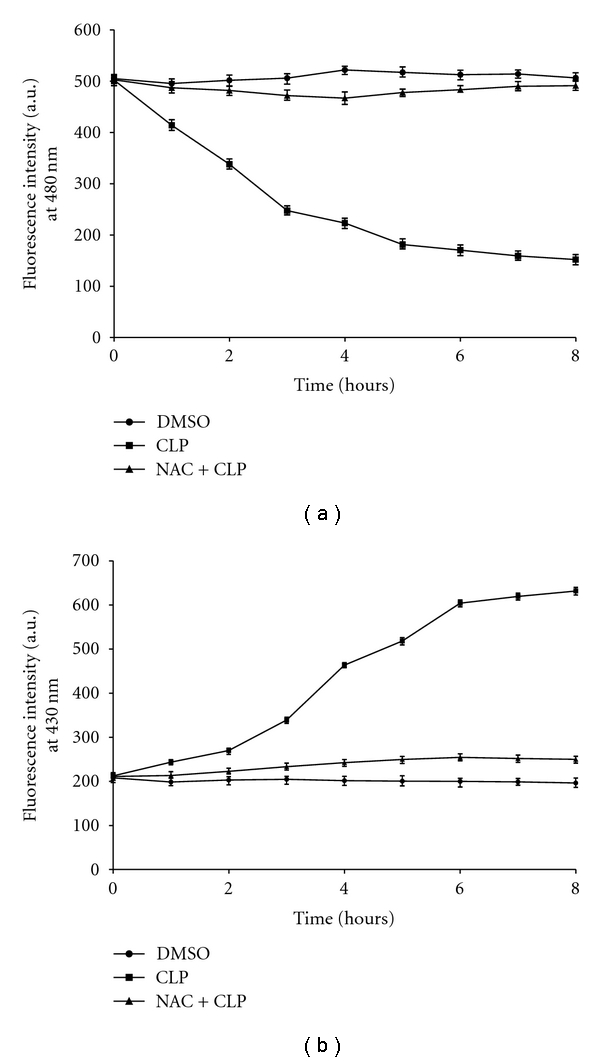
Determination of intracellular GSH level and level of lipid peroxidation in response to CLP treatment. (a) Level of intracellular GSH in treated and untreated *L. donovani *promastigotes. The intracellular GSH level was measured after treatment with 0.2% DMSO (closed circles), 20 *μ*M CLP (closed squares) and with NAC (20 mM) before treatment with CLP (closed triangles). (b) The level of fluorescent products of lipid peroxidation was measured after treatment of leishmanial cells with 0.2% DMSO (closed circles), 20 *μ*M CLP (closed squares) and with NAC (20 mM) before treatment with CLP (closed triangles). Data are represented as Mean ± SEM (*n* = 3).

One of the most important cellular defenses against intracellular oxidative stress is GSH, which plays a critical role in mediating apoptosis in eukaryotes, including leishmanial cells. GSH is an important molecule for protecting kinetoplastids from ROS or toxic compounds [4]. As shown in Figure 6(a), CLP causes a 49% decrease in GSH level after 3 h and the effect was more pronounced after 6 hrs treatment with CLP. When cells were preincubated with NAC (20 mM) for 1 h, followed by treatment with CLP, GSH level was protected significantly and tends to become normal.

Lipid peroxidation was assessed by measuring the total fluorescent lipid peroxidation products in leishmanial cells after treatment with CLP as described in Section 2. CLP treatment leads to an increase in lipid peroxides after 3 h of drug treatment and reached saturating level after 6 h. In the presence of 20 mM NAC, the level of fluorescent products decreased significantly (Figure 6(b)).

### 3.6. Inhibition of Autophagy Causes Upregulation of CLP-Induced Cell Death

**Figure 7:**
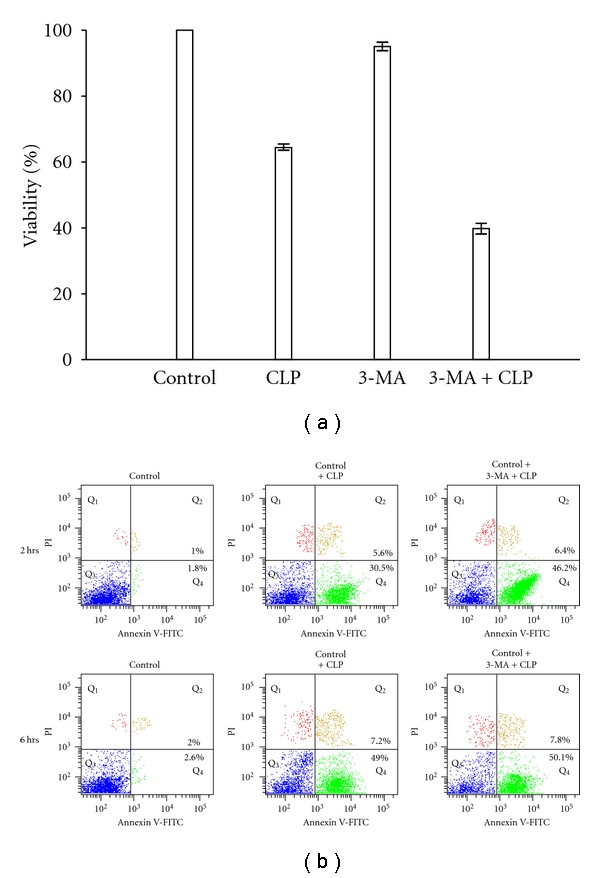
Effect of inhibition of autophagy by 3-MA, on CLP-induced cell death. (a) *L. donovani *promastigotes were treated with 0.2% DMSO, 20 *μ*M CLP, 10 mM 3-MA and 10 mM 3-MA prior to treatment with CLP for 2 h and percentage of cell viability was measured by MTT assay. Data are represented as Mean ± SEM (*n* = 3). (b) Flow cytometric analysis using annexin V and PI in FL-1 versus FL-2 channels. The cells were subjected to different treatments as shown in the figure for 2 h and 6 h, respectively, as described in Section 2. The annexin V positive cells (bottom right quadrant) denote apoptotic population.

Although treatment of *Leishmania* parasites with CLP shows initial features of autophagy, apoptosis-like cell death does occur at the later stage. To understand the relationship, if any, of the autophagic features with the cell death mechanism, we first determined the cell viability after CLP treatment when autophagy was inhibited by 3-MA. As evident from Figure 7(a), treatment with 20 *μ*M CLP for 2 h causes a 35% decrease in cell viability compared to control. However, when cells were pretreated with 3-MA, and then treated with 20 *μ*M CLP for 2 h, there was a 58% decrease in cell viability compared to control. Treatment with 3-MA only had no detectable effect on cell viability. These results suggest that pretreatment of *L. donovani *AG83 cells with 3-MA makes them more sensitive to CLP-induced cell death. This was further supported by the flow cytometric analysis. Cells were treated with 20 *μ*M CLP for 2 and 6 h with or without pretreatment with 3-MA and the percentage of apoptotic cells was determined by flow cytometric analysis after staining with annexin V-FITC and PI (Figure 7(b)). Externalization of phosphatidyl serine (stained by annexin V) and presence of impermeant cell membrane (negative PI staining) are hallmarks of PCD [13]. Flow cytometric analysis with annexin V/PI staining showed that when cells were exposed to CLP for 2 h, about 30.5% cells were annexin V positive (Figure 7(b)) but when cells were pretreated with 3-MA and then treated with CLP, about 46.2% cells were annexin V positive (Figure 7(b)). This suggests that inhibition of autophagy by 3-MA causes an increase the number of apoptotic cells. After 6 h of CLP treatment, 49% cells were annexin V positive and when pretreated with 3-MA before treatment with CLP for 6 h, about 50.1% cells were annexin V positive (Figure 7(b)). Interestingly, pretreatment with 3-MA and then adding CLP did not cause any formidable increase in the percentage of annexin V positive cells at 6 h time period. As inhibition of autophagy did not cause any significant increase in the cell death at 6 h time period, we surmise that the autophagic response may not influence the CLP-induced cell death mechanism at a later time period probably due to the prolonged intracellular stress which commits the cells to die.

### 3.7. RT-PCR Analysis

**Figure 8:**
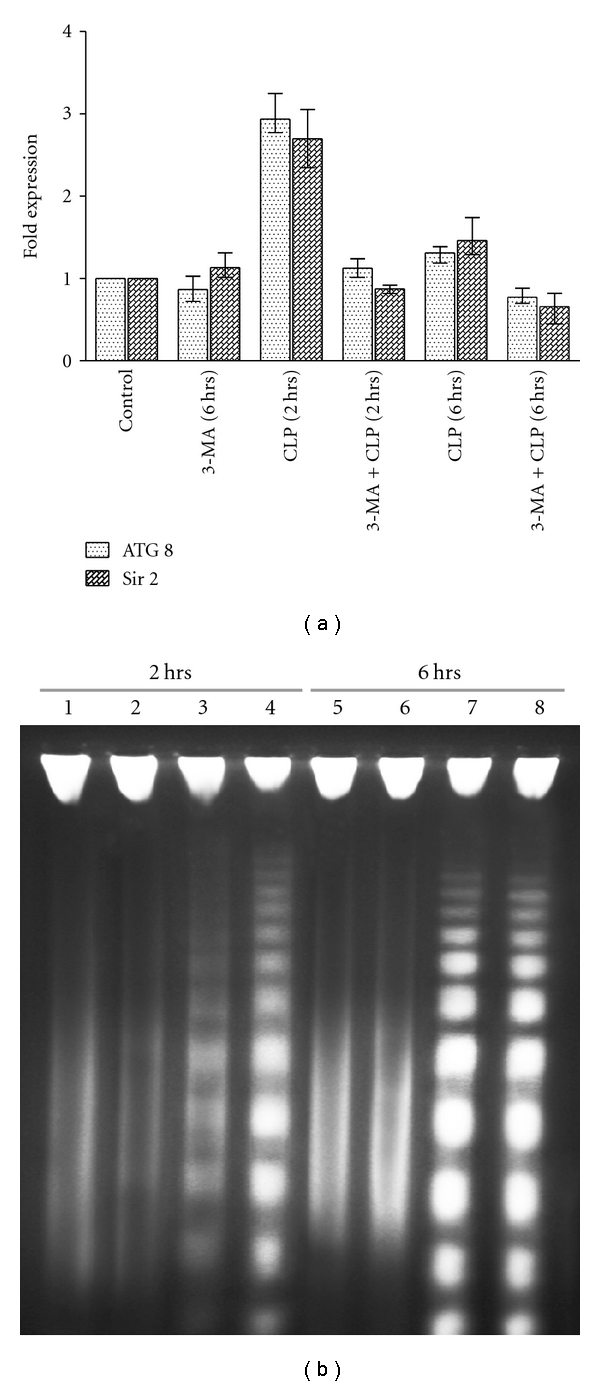
(a) Real-time PCR analysis for the expression level of ATG 8 and Sir2 genes from *L. donovani *promastigotes. The expression of ATG 8 and Sir2 were estimated relative to GAPDH in the treated samples compared with the untreated control. The fold expression was calculated as described in Section 2. The mean fold expression values are given in Table 2. Data are represented as Mean ± SEM (*n* = 3). (b) Fragmentation of genomic DNA in the presence and in the absence of 3-MA and CLP for different time periods. Genomic DNAs were isolated from *L. donovani* promastigotes after treatment with 0.2% DMSO alone for 2 h (lane 1), 10 mM 3-MA for 2 h (lane 2), 20 *μ*M CLP for 2 h (lane 3), and 20 *μ*M CLP for 2 h after pretreatment with 10 mM 3-MA (lane 4). Lanes 5–8, the same as lanes 1–4, respectively, but for 6 h.

To understand more clearly the role of the autophagic response in response to CLP treatment, we performed RT-PCR analysis with the autophagic gene ATG 8 [34] and Sir2 [35]. Sir2 is a member of silent information regulator family of genes [36] and has been implicated in lifespan extension along with autophagy [20]. Cytoplasmic Sir2 overexpression has been reported to promote survival of *Leishmania* parasites by preventing programmed cell death [36]. Thus, we investigated the effect on *Leishmania* Sir2 in the autophagic response induced by CLP. Treatment with CLP for 2 h causes marked increase in the mRNA level of ATG 8 (Figure 8(a)). CLP caused about 3-fold increase in the level of ATG 8 compared to untreated control at 2 h (Table 2). This confirms the involvement of autophagy in response to CLP treatment. However, cells pretreated with 3-MA before CLP treatment showed no significant change in ATG 8 mRNA levels confirming the inhibition of autophagy by 3-MA. Treatment with 3-MA only had no effect. Interestingly, treatment with CLP for 6 h did not show any significant change in the ATG 8 mRNA levels. This confirms the results of the flow cytometric analysis. Level of Sir2 was elevated after 2 h in response to CLP treatment (Figure 8(a)). CLP caused about 2.7-fold increase in the mRNA level of Sir2 compared to untreated control at 2 h (Table 2). This suggests that the autophagic response serves as a survival mechanism for the cells. However, pretreatment with 3-MA before addition of CLP caused a slight decrease in the mRNA level of Sir2. When cells were treated with CLP for 6 hrs, there was only 1.5-fold increase in the Sir2 mRNA level compared to untreated control suggesting the cells to be committed to death. Pretreatment with 3-MA caused a decrease in the Sir2 level compared to the untreated control cells. Taken together, the above results suggest that treatment of *L. donovani* AG83 promastigotes with CLP causes initial autophagic features as a survival mechanism which can be bypassed by employing specific inhibitor of autophagy (i.e., 3-MA). Moreover, the results also suggest that the survival mechanism cannot cope with the cellular stress at a later time period.

### 3.8. CLP Induces DNA Fragmentation in *L. donovani* AG83 Promastigotes

The internucleosomal DNA fragmentation by an endogenous nuclease (genomic DNA fragmentation) is considered as a hallmark of apoptotic cell death [3, 4, 37]. We observed internucleosomal DNA fragmentation in *L. donovani* AG83 cells in response to 20 *μ*M CLP treatment (Figure 8(b)). DNA fragmentation was significantly enhanced by combined treatment of 3-MA and CLP at 2 h compared to CLP alone (Figure 8(b), compare lanes 3 and 4). However, there was no significant difference at 6 h (Figure 8(b), compare lanes 7 and 8). This confirms the involvement of apoptosis-like cell death in *L. donovani *AG83 cells in response to CLP treatment which is augmented by inhibition of autophagy.

## 4. Discussion

*Leishmania donovani* is a unicellular protozoan parasite which causes visceral leishmaniasis worldwide. Treatment of leishmaniasis is unsatisfactory due to unavailability of effective vaccines and chemotherapy is still the mainstay for treating this dreaded disease. Moreover, emergence of resistance to traditional drugs has worsened the situation. Thus, there is an urgent need for new drug development and newer therapeutic strategies.

Cryptolepine is a naturally occurring indoloquinoline alkaloid which has been used as an antimalarial drug in Central and Western Africa. Cryptolepine has a broad spectrum of biological activity and has been reported to have anticancer activity [7]. In the present study, we have investigated the effect of cryptolepine on *L. donovani* AG83 promastigotes *in vitro*.

Our results show that CLP causes a decrease in the cell viability of *L. donovani* AG83 promastigotes in both time- and concentration-dependent manner. CLP causes an increase in cellular ROS production with concominant decrease in cellular GSH levels and increase in the level of lipid peroxidation. Also, CLP causes DNA fragmentation which is a hallmark of apoptosis. Altogether, these observations suggest the involvement of apoptosis-like cell death in response to CLP treatment. However, parasite ultra-structural studies by transmission electron microscopy led to some interesting observations. We observed multiple cytoplasmic vacuoles with normal nuclear architecture at an early stage after CLP treatment. This type of vacuolization was suggestive of autophagy [38, 39]. To understand the mechanism in more detail, we carried out staining with MDC which specifically labels autophagic vacuoles. It has been suggested previously that *Leishmania *contain a multivesicular tubule which is lysosomal in nature [31] and constitutes the endocytic compartment which is intimately involved with the autophagic pathway [32]. We observed clear MDC-positive vacuoles which colocalized with the multivesicular tubular compartment (FM4-64 positive) after treatment with CLP. These results confirm the involvement of autophagy in *L. donovani* AG83 promastigotes in response to CLP treatment.

To understand the relationship between autophagy induction and apoptosis-like cell death in more detail, we next carried out our study with 3-MA which is a specific inhibitor of autophagy [34]. MTT assay revealed that 3-MA and CLP cotreatment causes further decrease in the number of viable cells compared to CLP alone. This was further confirmed by flow cytometric analysis suggesting that autophagy serves as a survival mechanism and inhibition of autophagy can amplify the effect of CLP on *L. donovani* AG83 promastigotes. However, this effect is true only at an initial time period (2 h) as we observed no significant changes by inhibiting autophagy at a later time period (6 h). We surmise that at 6 hrs, CLP causes extensive damage to the cells which commits them to die rendering them unable to elicit the survival response.

Real-time PCR analysis revealed that there is a significant upregulation of ATG 8 transcript level in response to CLP treatment for 2 h though there was no significant change in the ATG 8 transcript level at 6 h compared to control untreated cells. This again confirmed the involvement of autophagy in the initial phase of CLP treatment. During past few years, the silent information regulator SIR2 protein family has attracted great interest due to its implication in an organism's life span extension [40]. It has been reported previously that Sir2 over expression promote survival of *Leishmania* parasites by preventing programmed cell death [36]. Also, transient overexpression of Sir2 has been clearly shown to stimulate the basal level of autophagy [20, 41]. Thus, we anticipated a role of Sir2 in CLP-induced cell death of *L. donovani* promastigotes. Rightfully, real-time PCR analysis revealed a significant upregulation in the Sir2 transcript level at 2 h after CLP treatment. This suggests that Sir2 may signal the onset of autophagy in response to CLP treatment. The fact that Sir2 can form molecular complex with several ATG genes and can deacetylate these proteins [41] explains the importance of Sir2 in the process.

From an evolutionary perspective, autophagy has been suggested to have originally evolved as a protective mechanism for unicellular eukaryotes against starvation and other environmental stresses [19]. Though the connection between autophagy and apoptotic cell death is not clear, autophagy has been reported to promote [42] or inhibit [33] apoptosis in cancer cells. There are also reports of autophagic cell death (type II cell death) in response to antimicrobial peptides in *L. donovani* [27] and in response to naphthoimidazoles in *T. cruzi* [34]. In the present study, we provide experimental evidence to show for the first time that autophagy represents a defense mechanism against CLP-induced cell death in *L. donovani* AG83 promastigotes. We have also shown that the morphological and biochemical changes associated with autophagy precede the onset of apoptosis-like cell death in these unicellular kinetoplastid protozoan parasites. Though dissection of the underlying molecular events is beyond the scope of this study, we surmise that Sir2 is an important candidate in the regulation of the autophagic response. Moreover, our findings also suggest that inhibition of autophagy by 3-MA can actually increase the effectivity of CLP-mediated cell killing. This finding can lead to development of new therapeutic strategies to combat leishmaniasis in future.

## Figures and Tables

**Table 1:** List of gene-specific primers used for real-time PCR analysis.

Target	Primer sequence
ATG 8	Forward: 5′-ATG TCT TCC AGA GTA GCT GGG-3′
	Reverse: 5′-ATT GAA GAG GTC GCT CAT GAG-3′

Sir2	Forward: 5′-TTT CGC TCA TCT GAC ACC GGG-3′
	Reverse: 5′-CCG CTG CCT TCT CCA GAC CAT-3′

GAPDH	Forward: 5′-AGA AGA CGG TGG ATA GTC ACT-3′
	Reverse: 5′-GCC ACA CCG TTG AAG TCT GAA-3′

**Table 2:** Fold of expression and corresponding fold change of ATG 8 and Sir2 genes relative to internal GAPDH control in treated samples compared with the untreated control.

Condition	ATG 8		Sir2	
	Mean fold expression	Fold change	Mean fold expression	Fold change
Control + 3-MA (6 hrs)	0.867571	~−1.2	1.132042	~1.1
Control + CLP (2 hrs)	2.936239	~3	2.697552	~2.7
Control + 3-MA + CLP (2 hrs)	1.126242	~1.1	0.873598	~−1.1
Control + CLP (6 hrs)	1.310307	~1.3	1.460229	~1.5
Control + 3-MA + CLP (6 hrs)	0.774112	~−1.3	0.659147	~−1.5

## Abbreviations

CLP: Cryptolepine
CPT: Camptothecin
Sir2: Silent information regulator 2
ATG 8: Autophagic gene 8
MTT: 3-(4,5-Dimethylthiazol-2-yl)-2,5-diphenyltetrazolium bromide
PI: Propidium iodide
ROS: Reactive oxygen species
DMSO: Dimethyl sulfoxide
NAC: *N*-Acetyl-L-cysteine
CM-H_2_DCFDA: 5-(and-6)-chloromethyl-2′,7′-dichlorodihydrofluorescein diacetate, acetyl ester
MDC: Monodansylcadaverine
PBS: Phosphate buffered saline
3-MA: 3-methyladenine
FM4: 64-*N*-(3-triethylammoniumpropyl)-4-(6-(4(diethylamino)phenyl)hexatrienyl)pyridinium dibromide
MVT: Multivesicular tubule.
